# Brain localization of Kaposi’s sarcoma in a patient treated by combination antiretroviral therapy

**DOI:** 10.1186/1471-2334-13-600

**Published:** 2013-12-21

**Authors:** Francesco Baldini, Andrea Baiocchini, Vincenzo Schininà, Chiara Agrati, Maria Letizia Giancola, Lucia Alba, Susanna Grisetti, Franca Del Nonno, Maria Rosaria Capobianchi, Andrea Antinori

**Affiliations:** 1 Clinical Department, National Institute for Infectious Diseases “L. Spallanzani” IRCCS, Via Portuense 292, 00149 Rome, Italy; 2 Department of Pathology, National Institute for Infectious Diseases “L. Spallanzani” IRCCS, Via Portuense 292, 00149 Rome, Italy; 3 Diagnostic Department, National Institute for Infectious Diseases “L. Spallanzani” IRCCS, Via Portuense 292, 00149 Rome, Italy; 4 Laboratory of Virology, National Institute for Infectious Diseases “L. Spallanzani” IRCCS, Via Portuense 292, 00149 Rome, Italy

**Keywords:** Kaposi’s sarcoma, HHV-8, HIV, Combination antiretroviral therapy, Central nervous system

## Abstract

**Background:**

Central nervous system is a very rare site of Kaposi’s sarcoma in acquired immunodeficiency syndrome. Kaposi’s sarcoma, a neoplasm of endothelial origin, occurs mainly in the skin, but can involve many tissues, especially in patients with a poor immunity. Combination antiretroviral therapy, highly active against human immunodeficiency virus type-1, has caused a dramatic reduction of cutaneous and visceral involvements. No report of central nervous system localization of Kaposi’s sarcoma is described since the introduction of combination antiretroviral therapy in the late 90’s.

**Case presentation:**

A 42 year-old Caucasian man affected by human immunodeficiency virus type-1 infection treated with combination antiretroviral therapy and showing relatively preserved immunity with low viral load presented gingival squamous cell carcinoma and visceral (lungs and lymph nodes) Kaposi’s sarcoma. Chemotherapy and radiotherapy were performed with improvement of both neoplasms. Afterwards, a magnetic resonance imaging showed focal lesions of the brain. Despite new chemotherapy and radiotherapy the patient died. Histology after autopsy revealed brain lesions due to Kaposi’s sarcoma with the detection of Human Herpesvirus 8 on tissue samples.

**Conclusions:**

This is the first report in the combination antiretroviral therapy era of a very rare complication of Kaposi’s sarcoma, such as that of brain localization, in a patient with a relatively good control of human immunodeficiency virus infection. Therefore, Kaposi’s sarcoma should be considered in differential diagnosis with other intracranial mass lesions that can occur in human immunodeficiency virus infected-patients focusing the issue of appropriate treatment for central nervous system involvement.

## Background

Since the first appearance, acquired immunodeficiency syndrome (AIDS) and Kaposi’s sarcoma (KS), a multicentric angioproliferative spindle cell tumor of endothelial origin, were associated [1]. KS can occur in many tissues, mainly in the skin, but also in visceral sites involving various organs like the gastrointestinal tract, the lungs, the lymph nodes, and the bone. For mucocutaneous KS an inspective, presumptive diagnosis is accepted [2]. DNA sequences of a new γ-herpesvirus were shown to be invariably present in KS lesions, but not in unaffected skin or in several other diseased tissues; hence the new virus was named KS-associated herpesvirus [3]. This virus belongs to the herpesviridae family, and is also taxonomically classified as Human Herpesvirus 8 (HHV8). The causal association between HHV8 and KS has been subsequently proven by unequivocal evidence, including the overlapping global patterns of disease incidence and viral seroprevalence [4] and gave a support for the diagnosis. However, for diagnosis of certain localizations, other than mucocutaneous forms like lymph nodes and bone, histological examination remains necessary, because a differential diagnosis between KS and other diseases is required. Currently KS continues to represent one of the most relevant cause of AIDS-defining events, although less frequently since the introduction of combination antiretroviral therapy (cART), which caused a dramatic reduction of cutaneous manifestation and visceral involvements [5]. Central nervous system (CNS) is a very rare site of KS, both in the human immunodeficiency virus type 1 (HIV-1) [6] and in the general population setting [7]. Isolated histological cases of brain KS in HIV-infected patients were reported at the beginning of the AIDS era and before the introduction of cART. Some were fully characterized cases, others were found in CNS diseases studies or in retrospective autoptic series of HIV-infected patients [8-13]. Up to now, no report of brain localization is described in cART era.

We here describe the case of a HIV-1-infected patient with KS involvement of the brain, focusing on histopathological, virological and radiological features.

## Case presentation

A 42 year-old Italian man diagnosed with HIV-1 infection since 1993, was admitted at our hospital in April 2011 complaining about an acute pain at the right zygoma. At medical examination he had a vegetated lesion of the gingiva, arising from the superior maxilla, and an enlargement of right supraclavear lymph nodes. Examination of the other organs was normal. CD4+ cell count was 243/mm^3^ and HIV-1 RNA in plasma was 68 copies/mL.

At clinical history, cutaneous and pulmonary KS had been diagnosed in 1993 and at that time successfully treated with interferon alfa and chemotherapy. CD4+ cell count nadir was 52/mm^3^ and hepatitis C virus coinfection was documented. In recent years, only occasional bacterial pneumonia had occurred. cART was started in 1997 and changed to various regimens up to 2008, since when the current cART based on co-formulated tenofovir-emtricitabine, raltegravir, and darunavir/ritonavir had been prescribed. This salvage therapy was chosen after HIV genotypic resistance test that documented mutation associated with multidrug resistance. CD4+ cell count had stably risen above 200/mm^3^ since December 2008 and HIV-1 RNA level was constantly below 500 copies/mL since October 2009.

During hospitalization a total body computerized tomography (CT) showed multiple nodular lesions in both lungs, minimal right pneumothorax, pathological soft tissue in the right maxillar sinus, and enlargement of supraclavear lymph nodes. CT scan of the brain was normal. Biopsy specimen from gingival lesion revealed squamous cell carcinoma, while tissue taken from supraclavear lymph node showed histological features of KS. HHV8-DNA by polymerase chain reaction (PCR) was negative on blood. Four cycles of chemotherapy using liposomial doxorubicin (20 mg/m^2^) were performed, and after only two cycles an improvement of CT findings was documented. Then, ten sessions of radiotherapy on maxillofacial site for carcinoma were performed (total dose 30 Gy).

**Figure 1:**
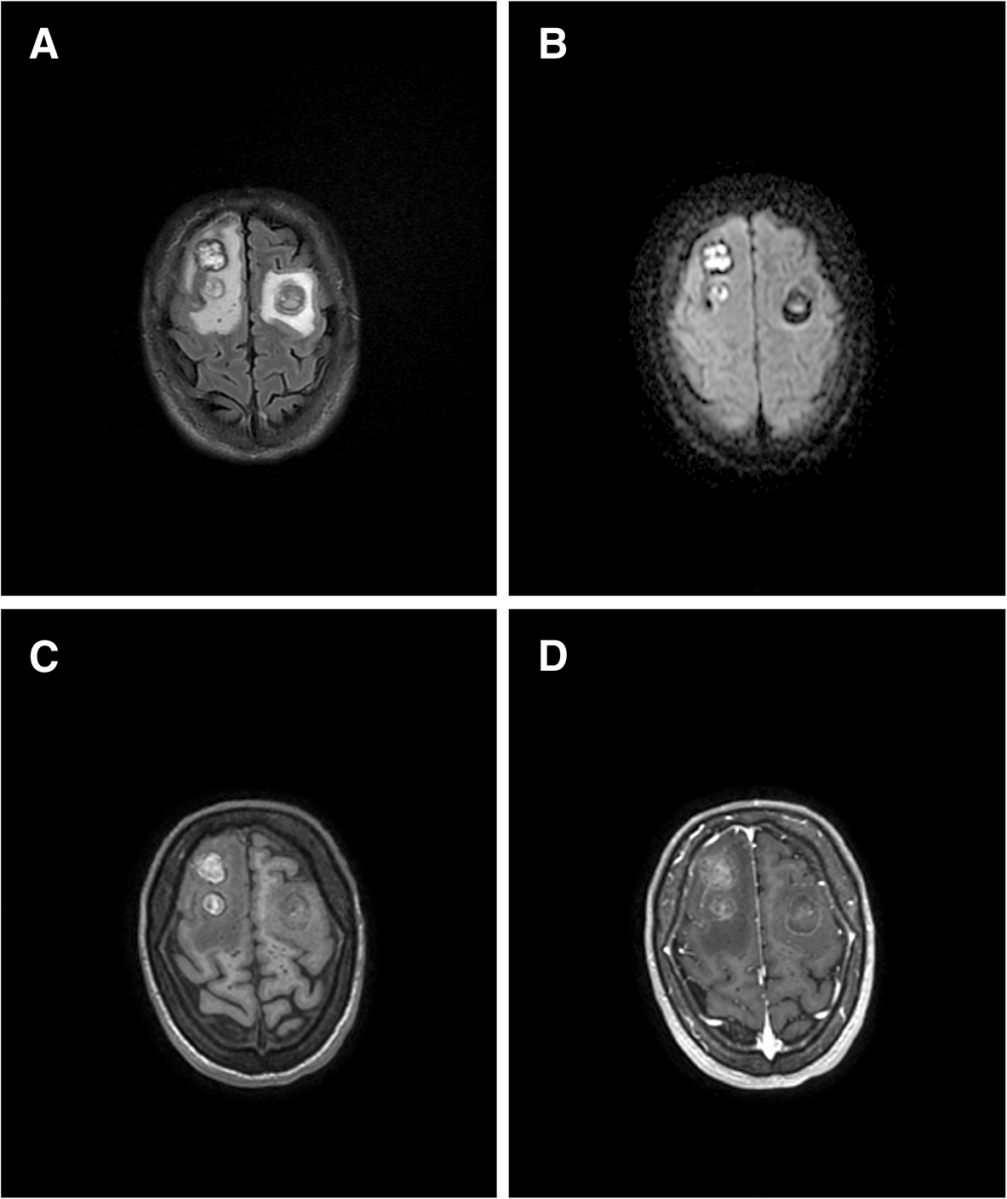
**Magnetic Resonance Images (MRI) of the brain at control after 26 days of anti-*Toxoplasma *treatment. A**. On Axial FLAIR T2-weighted images the lesions are hyperintense, with hypointense halo and surrounding edema. **B**. Diffusion-weighted imaging of the lesions showed restricted diffusion. **C**. On unenhanced Axial T1-weighted images the lesions signal is heterogeneous. **D**. On enhanced Axial T1-weighted images faint lesions enhancement are depicted.

In October 2011, the patient showed bilateral spontaneous pneumothorax and was treated with chest drainage. After 25 days, he presented generalized seizures. A magnetic resonance imaging (MRI) of the brain showed three focal brain lesions, two that involved the right frontal lobe (20 and 14 mm) and one the right cerebellum hemisphere (16 mm). On FLAIR T2-weighted images the lesions of the right frontal lobe were hyperintense, with hypointense halo and surrounding edema. Diffusion-weighted imaging (DWI) showed restricted diffusion and low apparent diffusion coefficients (ADCs) in the lesions (range avg 0,458-0,566x10(-3)mm(2)/s). A new total body CT displayed a worsening of the previously described pulmonary lesions and the development of new intestinal lesions. A multi-disciplinary consultation excluded the possibility to carry out a cerebral biopsy, because of the lesion high risk of bleeding. CD4+ cell count was 142/mm^3^ and HIV-1 RNA in plasma was 225 copies/mL. HHV8-DNA by PCR on blood was repeatedly negative and IgG anti-*Toxoplasma gondii* antibodies were absent. Nevertheless, empiric anti-*Toxoplasma* treatment with pyrimetamine plus sulfadiazine, combined with steroids and phenobarbital, was prescribed. Brain MRI after 26 days of treatment showed worsening of the CNS disorder, with the appearance of a new lesion in the left frontal lobe displaying the same radiological characteristics. There was no change in signal intensity or size of the lesions already described in the previous examination. The lesions appeared as inhomogeneous masses, hyperintense on T1- and on T2-weighted images (Figure 1A, B, C), with halo hypointense sign and surrounding edema, minimal mass effect and faintly ring enhancement. DWI of the lesions showed restricted diffusion (Figure 1D) and lower ADC (avg 0,285×10(-3)mm(2)/s). Thallium-201 single-photon emission computed tomography (^201^Tl SPECT) of the brain did not show an increased uptake in the brain. 18 F-2-Fluoro-2-deoxy-d-glucose (FDG) positron emission tomography (PET) of the neck, thorax and abdomen showed hyperperfused tissue in the right cervical lymph nodes, skeleton, right adrenal gland, lungs, right maxillar sinus, duodenum. On the 1st of December a cycle of paclitaxel (100 mg/m^2^) was administered, and then ten sessions of whole brain radiotherapy were performed (total dose 30 Gy). At the end of December a massive right spontaneous pneumothorax occurred and pleural drainage was necessary again. The patient died on January 6th, 2012.

**Figure 2:**
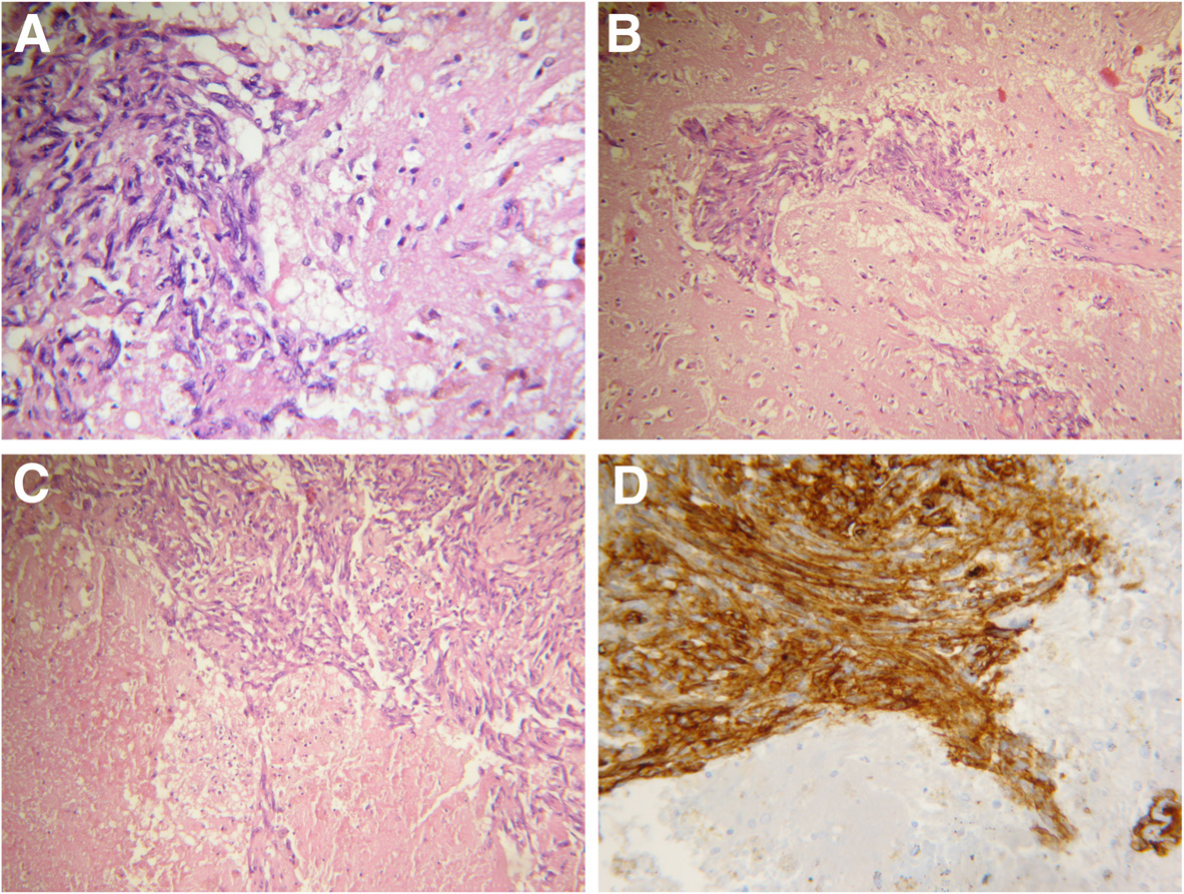
**Histological findings in blood-borne brain lesion. A**. The multiple hemorrhagic nodules showed whorls of spindle-shaped cells and neovascularization with small-vessel proliferation characteristic of Kaposi’s sarcoma (hematoxylin-eosin stain; original magnification, ×400). **B**. Tumour cells invaded brain parenchyma through perivascular space (hematoxylin-eosin stain; original magnification, ×200). **C**. Neoplastic nodules showed hemorrhagic necrosis with pigmented macrophages (hematoxylin-eosin stain; original magnification, ×200). **D**. The neoplastic cells show strong cytoplasmic staining for CD34 (immunoperoxidase on a paraffin embedded section, original magnification, ×400).

A full autopsy, including neuropathological examination, was performed. Tissue samples were taken from all organs. KS was found in the lungs, the ampulla of Vater, the pancreas, the duodenum, the adrenal gland and the cerebrum. Macroscopic examination revealed hemorrhagic nodules, variegated in appearance, with cystic degeneration in the organs involved. Multiple bilateral areas of consolidation with cavitation and necrotizing nodular lesions were present in the lungs. Cavities were extended to the pleura. Coronal section of the brain showed multiple hemorrhagic nodules with swelling of the cerebrum without displacement of midline structures. Lesions were localized in the frontal lobes at the gray/white matter interface, near the falx and in the cerebellum. Microscopic appearance of the tumor nodules was composed of monomorphic spindle cells arranged in ill defined fascicles (Figure 2A) and separated by slit like vessels containing erythrocytes. Brain parenchyma of both cerebellar and cerebral hemispheres was invaded by the tumor cells directly or through the perivascular space (Figure 2B). Neoplastic nodules showed hemorrhagic necrosis with pigmented macrophages (Figure 2C). Immunohistochemical staining of the tumour cells revealed strong expression of CD34 (Figure 2D). Real time PCR assays [14] revealed HHV8 DNA sequences in the tissue samples from the duodenum, the lungs and the brain. Quantitative evaluation of HHV8 DNA in the lungs, the Vater’s papilla and the brain showed: 15872 copies/10^6^ cells, 7789 copies/10^6^ cells and 2316 copies/10^6^ cells respectively.

## Conclusions

This case report describes an HIV-1-infected patient presenting brain localization of KS, despite previously administered chemoterapy and cART treatment. Disease localization was documented by accurate histological, virological and radiological examination with MRI and DWI. At first evaluation of the patient, absence of cutaneous neoplastic lesions, immunological status with CD4+ cell count stably above 200/mm^3^, HIV-1 RNA of 68 copies/mL, negative HHV8 PCR in the peripheral blood, and diagnosis of concurrent gingival carcinoma made the diagnosis of KS unlikely.

In the pre-cART era, HIV-1-infected patients with KS typically had a low CD4 cell count and a high HIV viral load. Since the introduction of cART, the incidence of KS has decreased [5] and survival improved [15,16]. Further, it is well documented that cART, especially HIV protease inhibitors, can improve KS lesions with or without use of liposomal anthracycline [15,17]. However, unusual cases of cutaneous HIV-associated KS occurring even during stable immunologic rescue and HIV-RNA suppression in plasma are reported [18,19].

Our patient was cART-experienced, with genotypic multi-drug resistance in HIV. Despite the high-level resistance, re-optimized cART allowed to obtain stable low-level viremia in plasma close to 50 copies/mL cut-off. Biopsy of the lymph node revealed histologic KS and allowed starting chemotherapy with improvement of visceral lesions. At the onset of neurological symptoms, brain mass lesions at MRI were detected. Radiologic features of intracranial KS have only rarely been described; intracranial masses of the patient had a vascular component, and appeared as a inhomogeneous mass with hyperintense signal on T1- and on T2-weighted images, with surrounding edema, minimal mass effect and with faintly ring enhancement. The hyperintensity on T1-weighted images probably was due to abnormally increased blood vessels with slow intra-lesional flow. The low ADC value of KS lesions might be due to its histological architecture made of high tumor cellularity and relatively large vascular spaces. Although no studies have elucidated the direct relationship between blood flow velocity and vascular spaces size we hypothesise that blood velocity in KS lesions is low because of the large vascular spaces, and that this may determine the increased signal strengths and lower ADCs on DWI. FDG-PET of the body only showed the suspicion of neoplastic disease in various tissues, but FDG-PET of the brain unfortunately was not performed. Only autopsy specimens allowed obtaining the diagnosis of KS, that was confirmed by the detection of HHV8-DNA in the brain tissues. In HIV-infected patients, differential diagnosis of mass lesions is required. Mass algorithms in the pre-cART era were well defined, but in the cART era, when the epidemiology of opportunistic infections changed, are lacking. Brain biopsy, if feasible, is no longer mandatory for the diagnosis of mass lesions in HIV-patients [20], however histological examination in some cases is still the only way to obtain a definitive diagnosis.

Only few histologically proven cases of KS metastatic to the brain in HIV-1 patients have been described: up to now Gorin *et al.* reported two cases at the beginning of the AIDS era [8], and Buttner *et al.* described in 1997 a patient who had never taken cART [9]. Two more cases were published by Levy *et al.*[10] and one was observed by Post *et al.* in CNS diseases studies performed in the 80’s [11]. In a morphological analysis of brains from 100 AIDS patients observed in the course of 1987–1995 years, Mossakowski *et al.* found one case of cerebral KS [12], so did Jellinger *et al.* in a retrospective study on 450 consecutive autoptical AIDS cases between 1984 and 1999, only in the cohort of 1984–1992 [13]. Most of these cases were observed in advanced patients with other concomitant AIDS-related events and none occurred during antiretroviral treatment. Moreover, in our patient cART was mainly effective, with low plasma viral copies and partial immune recovery.

The cause of brain involvement in this patient despite an initial improvement of visceral lesions with the appropriate treatment [21], is difficult to explain. Furthermore, it is well documented that positive HHV8 DNA on peripheral blood or a CD4 level below 200/mm^3^ are risk factors of poor evolution of KS [22], but the patient had a stable immunity and a persistent negative blood HHV8 DNA. Despite incomplete suppression of HIV-1 replication, cART was effective to prevent the development of opportunistic infections in all the clinical history of the patient. We know that HIV-1 plays a role on the KS diffusion through the production and the release of the HIV-1 Tat protein, a KS progression factor, and that cART has a documented direct anti-angiogenetic effect [23,24]. In this case, independently of the CD4 cell count, the incomplete suppression of HIV-1 could have enhanced the diffusion of KS, producing an increased release of the HIV-1 Tat protein.

This case describes a rare complication of KS, never reported in the cART era, and raises a question about the diagnosis and the treatment suggesting that KS should be considered for the differential diagnosis with other intracranial mass lesions that can occur in HIV patients and focusing on the problem of appropriate treatment for CNS involvement.

The case described was approved by an internal revision group at the Direction of Clinical Department.

### Consent

Written informed consent was obtained from the wife of the patient for publication of this case report and any accompanying images. A copy of the consent form is available for review by the Editor of this journal.

## Competing interests

The authors declare that they have no competing interests.

## Authors’ contributions

FB: study concept, acquisition, analysis and interpretation of data, drafting and critical revision of the manuscript. AB, VS, CA, FDN, MRC: acquisition, analysis and interpretation of data, drafting of the manuscript. MLG, AA: study concept and design, drafting and critical revision of the manuscript. LA, SG: acquisition of data, critical revision of the manuscript. All the authors approved the final version of the manuscript.

## Pre-publication history

The pre-publication history for this paper can be accessed here:

http://www.biomedcentral.com/1471-2334/13/600/prepub
